# Whole-body vibration exercise for low back pain: A meta-analysis protocol of randomized controlled trial

**DOI:** 10.1097/MD.0000000000012534

**Published:** 2018-09-21

**Authors:** Yi-Li Zheng, Zhi-Jie Zhang, Meng-Si Peng, Hao-Yu Hu, Ju Zhang, Xue-Qiang Wang

**Affiliations:** a Department of Sport Rehabilitation, Shanghai University of Sport; b Department of Rehabilitation Medicine, Shanghai Shangti Orthopaedic Hospital, Shanghai; c Rehabilitation Therapy Center, Henan Province Orthopedic Hospital, Luoyang, China.

**Keywords:** low back pain, meta-analysis, physical therapy, whole-body vibration exercise

## Abstract

**Background:**

Low back pain (LBP) affects about 80% of the population at some time in their lives. Exercise therapy is the most widely used nonsurgical intervention for low back pain in practice. Thus, whole-body vibration (WBV) exercise is becoming increasingly popular in relieving musculoskeletal pain and in improving function. However, the efficacy of WBV exercise is not without dispute for low back pain. This meta-analysis, with its comprehensive and rigorous methodology, will provide better insight into this problem.

**Methods:**

Published articles will be identified using electronic searches from 1950 to 2018. The Cochrane Library, PubMed, Web of Science, Embase, CINAHL-Ebsco, PEDro, and China Biology Medicine will be searched for randomized controlled trials. Studies without a parallel comparison group will be excluded. Two reviewers will independently select relevant studies that investigate on WBV exercise for patients with LBP. Data extraction will be done independently by the same 2 reviewers who selected the studies. The primary outcome will be to assess pain, back-specific disability index, and adverse effect. Continuous data will be pooled by calculating the mean difference using the random-effects model.

**Results:**

The results of the final meta-analysis will provide an evidence of WBV exercise for low back pain.

**Conclusion:**

This meta-analysis will provide a detailed summary of the evidence on the effects of WBV exercise on patients with LBP, in comparison with general exercise or without treatment. This review will benefit to researchers and policy makers who are interested in the treatment of LBP by WBV exercise.

## Abstract 2

Supplemental Digital Content is available in the text

## 1 Introduction

Low back pain (LBP) is one of the leading causes of disability and remains a main public health concern in many developed countries.^[1,2]^ Currently, the prevalence of LBP is reported to be 84% and that of chronic LBP is about 23%, with 12% of patients becoming disabled because of LBP.^[3]^ The effect of LBP goes beyond the pain itself and could be profound and serious, especially with LBP frequently resulting in indirect costs, loss of productive capacity, and increase in risk of suffering from other medical issues.^[4,5]^ In Australia in 2001, the total cost of LBP in 2001 was AUS$9.17 billion, whereas the direct health care costs reached only AUS$1.02 billion.^[6]^ In the United States, the total direct and indirect expenses for LBP treatment are approximated to be more than $100 billion every year.^[7]^

Most clinical practices^[3,8,9]^ recommend exercise intervention as an efficient solution to improve back function and decrease pain in patients with LBP. Whole-body vibration (WBV) exercise is a popular method of reducing pain intensity and improving function and quality of life.^[10]^ WBV exercise involves performing immobile standing or conducting dynamic exercises through a vibratory platform.^[11,12]^ Vibratory stimulation plus exercise intervention may complement standard physical rehabilitation programs for LBP.^[13]^

The WBV exercise is currently being promoted as a treatment for patients with LBP.^[13–21]^ A theoretical basis explains the effect of WBV exercise on the treatment of LBP. First, positive correlations exist between core muscle inactivity and pain intensity and the function of patients with LBP.^[22–24]^ WBV exercise could activate muscle fibers and strengthen core stability muscles to improve back function in patients with LBP.^[25–27]^

Second, paravertebral muscle spasm occurs in most patients with LBP, and WBV exercise may relieve pain by relaxing the paravertebral muscles.^[21]^ Third, proprioception deficits of the lumbosacral region often lead to spine dysfunction and instability in patients with LBP. WBV exercise could improve proprioceptive function by activating the proprioceptors of patients with LBP.^[14,28]^

Despite these benefits of WBV exercise, its exact effect on LBP has yet to be fully understood. Research suggests that WBV exercise could alleviate pain intensity and improve function in patients with LBP.^[18,19,21]^ Conversely, several articles report that WBV exercise is not useful for patients with LBP and that exposure to vibrations is traditionally considered to be harmful.^[29–31]^ Scientific evidence on WBV exercise for LBP should be ascertained to save time and reduce the burden of patients with LBP and their families. Therefore, a meta-analysis of all randomized controlled trials (RCTs) is conducted in this study to assess the effect of WBV exercise on patients with LBP. The results are then compared with those of general exercise or the case without intervention.

## 2 Methods

### 2.1 Study registration

The meta-analysis will be performed and reported in accordance with the PRISMA-P 2015 statement^[32,33]^ (Supplemental file 1). The protocol is registered on the international prospective register of systematic reviews (PROSPERO registration number: CRD42012003505. Available at: http://www.crd.york.ac.uk/prospero/).

### 2.2 Search strategy

Relevant articles will be identified by electronically searching the following data sources (from 1950 to 2018): PubMed, Web of Science, Embase, the Cochrane Library, CINAHL-Ebsco, PEDro, and China National Knowledge Infrastructure. The search will be limited to RCTs, and no language restriction will be imposed.

The details of the PubMed search are provided in Table 1. The duplicates identified after the database search will be removed. We will manually scan reference lists to identify other relevant articles from several sports medicine journals.

**Table 1:**
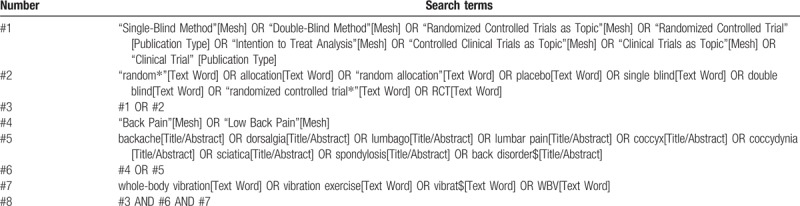
Search strategy for PubMed.

### 2.3 Criteria in considering studies for this review

#### 2.3.1 Types of studies

Published RCTs will be included. No restrictions on language used or trial date will be imposed.

#### 2.3.2 Types of participants

Articles involving both male and female participants (older than 18 years) will be included.

#### 2.3.3 Types of interventions

Studies that compare intervention groups receiving WBV exercise and control groups receiving general exercises, such as strength and stretching exercises, or no treatment will be included. Table 2 reports model of pairwise comparisons which would be used to determine studies criteria. WBV exercise is a training method in which an individual performs physical activities while on a machine that sends vibration signals to the body.

**Table 2:**
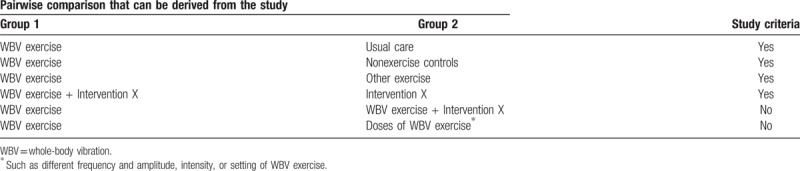
Model of pairwise comparisons that will be used to determine study criteria.

#### 2.3.4 Types of outcome measures

Outcome measures will be assessed on the basis of the analyses of individual patient data from published studies. Outcomes will be recorded in 3 time periods: short term (not longer than 3 months), intermediate (6 months), and long term (1 year or more).

The primary outcomes include the following:

1. Pain intensity: Pain intensity will be measured with a pain numeric rating scale (NRS) and a visual analog scale (VAS). The NRS is an 11-point (from 0 to 10) scale for patient self-reporting of pain. The VAS is marked as “no pain” (score 0) and “unbearable pain” (score 10) from left to right.
2. Back-specific disability index: The back-specific disability index will be determined from the responses in the Oswestry disability index (ODI) obtained using the Roland–Morris disability questionnaire (RMDQ). The ODI covers 10 items, with the value of 0 representing no disability and the value of 100 representing the maximum disability possible. The RMDQ score ranges from 0 to 24. A high score equates to poor back function.
3. Adverse event: Reports of adverse events associated with WBV exercise will be considered.

The secondary outcomes include the following:

1. Global improvement: Global improvement and quality of life will be measured with questionnaires such as short form-36 (SF-36).
2. Return to work/absenteeism
3. Depression and anxiety.

### 2.4 Selection of studies

**Figure 1:**
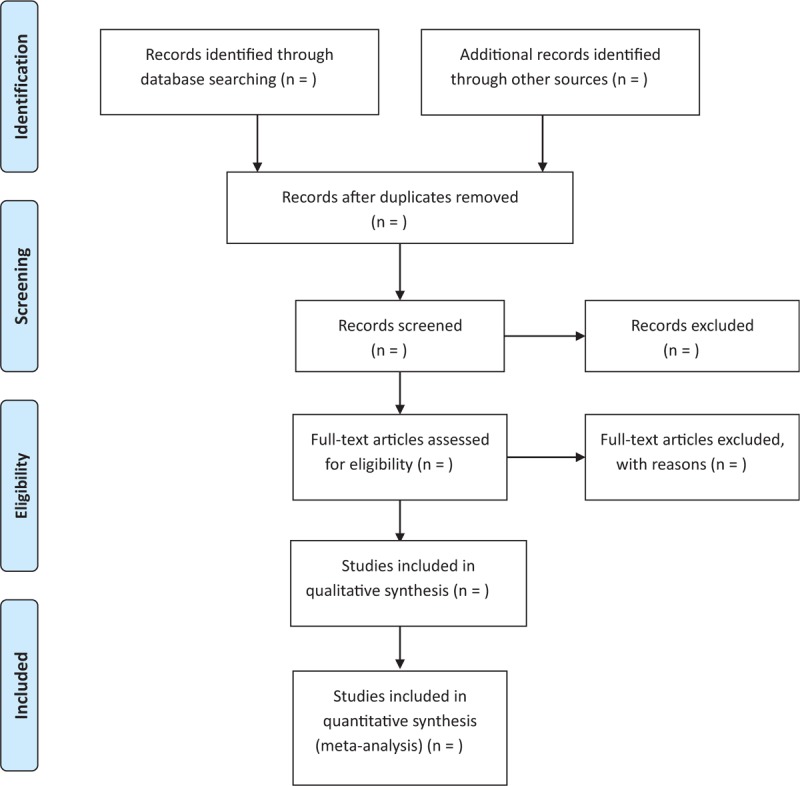
Flow chart of the study selection procedure.

All citations identified through the performed search will be downloaded into a reference manager database. Two authors with familiarity with the cited authors and publication journals will independently screen the studies to be included using prespecified criteria. If the abstract of a study fails to meet the selection criteria, the study will be immediately excluded. Otherwise, the full paper will be retrieved. Two authors using the same selection criteria will conduct the abstract screening, read the full papers, and make final selection decisions. Any discrepancies will be resolved through discussion; if necessary, a 3rd reviewer will step in to resolve disagreements. Figure 1 shows the flow chart of the study selection procedure.

### 2.5 Data extraction

A standardized form will be used to extract data from the included studies. The following data will be extracted: study design, patient characteristics (e.g., number of participants and age), study characteristics (e.g., country where the study was conducted and year), description of the experimental and control interventions, duration of the follow-up, types of assessed outcomes, and time points. Data will be extracted independently by the same authors who conducted the selection of studies. Any disagreements will be discussed, and an arbiter will be consulted when necessary. Data related to the primary outcomes will be assessed for inclusion in the meta-analyses, and the final value scores (means and standard deviations [SDs]) will be extracted.

### 2.6 Dealing with missing data

We will contact the authors of the included studies if means or SDs are not reported. We will approximate the means and SDs for the data reported in a graph rather than a table. If a study reports median, interquartile range (IQR), standard errors, 95% confidence interval (CI), or *P*-values without SDs, we will transform these values into means or SDs. For example, if continuous data are shown as IQR or median, we will estimate the median to be equivalent to the mean, and we will approximate the SD from the IQR data using the equation.

### 2.7 Quality assessment of included studies

We will use the Cochrane Collaboration recommendations^[34]^ to evaluate the quality of all included studies. The following information will be assessed: random sequence generation, allocation concealment, blinding of participants and personnel, blinding of outcome assessment, incomplete outcome data, selective reporting, and other biases. Two authors will evaluate the methodologic quality of all included studies. An arbiter will be consulted to address any disagreements.

Regardless of the amount of data available for the quantitative analyses and data summary, the overall quality of the evidence for each outcome will be assessed. The Grading of Recommendations Assessment, Development, and Evaluation approach^[35]^ will be used to assess the overall quality of the evidence by employing the adopted version of the criteria advocated by the Cochrane Back Review Group.^[36]^ The factors that may decrease the quality of the evidence are the study design and risk of bias, inconsistency of the results, indirectness (not generalizable), imprecision (sparse data), and others (e.g., reporting bias). The quality of the evidence for a specific outcome will be reduced by a level on the basis of the performance of the studies against these 5 factors.

### 2.8 Statistical analysis

#### 2.8.1 Data analysis

Relative risk will be used to analyze dichotomous outcomes. The mean difference or the standardized mean difference will be used to analyze continuous outcomes. The standardized mean difference will be selected if different outcomes or units are used to measure the outcomes. Mean difference will be chosen if the same outcome or unit is used to measure outcomes. Uncertainty will be expressed with 95% CI. Outcome measurements from individual trials will be combined through meta-analysis, where possible (intervention, outcomes and clinical comparability of patients with LBP between trials), using a random-effect model. The results will be qualitatively described from clinically comparable studies in the review if the outcome is not possible for meta-analysis.

#### 2.8.2 Assessment of heterogeneity

The Chi-squared and *I*^2^ tests will be used to assess the heterogeneity of the included articles. If *I*^2^ < 50% and *P* > .10 (Chi-squared test), the heterogeneity will be regarded as acceptable. The *P*-value of .10 of the Chi-squared test and *I*^2^ > 50% indicate a significant statistical heterogeneity.

#### 2.8.3 Assessment of publication biases

With sufficient data, Egger regression test will be performed to assess potential publication bias using Stata 12.0.

#### 2.8.4 Subgroup analysis

With adequate data, we will perform the following subgroup analyses:

1. Types of control groups (e.g., general exercise, placebo, wait-and-see groups)
2. Types of patients (e.g., acute, subacute, chronic LBP)
3. Duration of follow-up (i.e., short, medium, and long term)
4. Differential application of WBV exercise (e.g., different frequencies, amplitudes).

## 3 Discussion

Many different types of exercise programs are used to relieve pain and improve back function for patients with LBP. Recently, WBV exercise has become increasingly popular in relieving LBP and improving function and quality of life. Although WBV exercise has a theoretical basis in the treatment of LBP, its effect on the improvement of pain and function is controversial.

A previous systematic review^[37]^ included 3 RCTs (published between 2002 and 2005) and concluded the need to perform a strict study designed to confirm the effect of WBV exercise as a safe and high-quality intervention for LBP. However, the meta-analysis only focused on the result description of the included articles and on the qualitative synthesis rather than on the meta-analysis. Another systematic review^[38]^ covering 27 studies reported that exposure to WBV increases the risk of LBP and sciatica. However, this systematic review focused on evaluating the association occupational exposure to WBV and LBP. Occupational exposure to WBV is different from WBV exercise, which entails long WBV exposure time and regular WBV patterns (e.g., frequency and amplitude). For example, truck drivers and farmers are often exposed to WBV while driving trucks and operating farm equipment.

At present, no systematic review and meta-analysis has been performed to determine the efficacy of WBV exercise in improving back function and reducing pain in patients with LBP. In addition, whether WBV exercise is useful in patients with LBP remains unclear. Therefore, the aim of our meta-analysis is to ascertain the effectiveness of WBV exercise in improving pain and back function in patients with LBP, whether WBV exercise offers more beneficial effects than general exercise for patients with LBP, and the adverse effects associated with WBV exercise. This meta-analysis will benefit researchers and policy makers who are interested in the treatment of LBP via WBV exercise.

## Author contributions

**Conceptualization:** Yili Zheng, Zhijie Zhang, Xueqiang Wang.

**Data curation:** Yili Zheng, Mengsi Peng, Haoyu Hu.

**Formal analysis:** Zhijie Zhang, Mengsi Peng, Haoyu Hu.

**Funding acquisition:** Haoyu Hu, Xueqiang Wang.

**Investigation:** Zhijie Zhang, Haoyu Hu.

**Methodology:** Yili Zheng, Haoyu Hu, Xueqiang Wang.

**Project administration:** Mengsi Peng, Haoyu Hu.

**Resources:** Mengsi Peng, Juan Zhang.

**Software:** Zhijie Zhang, Juan Zhang.

**Supervision:** Yili Zheng, Mengsi Peng, Juan Zhang, Xueqiang Wang.

**Validation:** Mengsi Peng, Juan Zhang.

**Visualization:** Mengsi Peng, Juan Zhang.

**Writing – original draft:** Yili Zheng, Zhijie Zhang, Xueqiang Wang.

**Writing – review & editing:** Zhijie Zhang, Xueqiang Wang.

## Supplementary Material

Supplemental Digital Content
